# Physical activity after commitment lotteries: examining long-term results in a cluster randomized trial

**DOI:** 10.1007/s10865-018-9915-x

**Published:** 2018-02-26

**Authors:** Koen van der Swaluw, Mattijs S. Lambooij, Jolanda J. P. Mathijssen, Maarten Schipper, Marcel Zeelenberg, Stef Berkhout, Johan J. Polder, Henriëtte M. Prast

**Affiliations:** 1 Tilburg School of Social and Behavioral Sciences, Tranzo Scientific Center for Care and Welfare, Tilburg University, PO Box 90153, 5000 LE Tilburg, The Netherlands; 2 Department of Quality of Care and Health Economics, Center for Nutrition, Prevention and Health Services, National Institute of Public Health and the Environment (RIVM), PO Box 1, 3720 BA Bilthoven, The Netherlands; 3 Department of Statistics, Informatics and Modelling, Center for Nutrition, Prevention and Health Services, National Institute of Public Health and the Environment (RIVM), PO Box 1, 3720 BA Bilthoven, The Netherlands; 4 Department of Social Psychology, Tilburg School of Social and Behavioral Sciences, Tilburg University, PO Box 90153, 5000 LE Tilburg, The Netherlands; 5 Department of Marketing, School of Business and Economics, VU Amsterdam, De Boelelaan 1105, 1081 HV Amsterdam, The Netherlands; 6 Department of Quality Management, High Five Health Promotion, Schinkeldijkje 18, 1432 CE Aalsmeer, The Netherlands; 7 Department of Finance, Tilburg School of Economics and Management, Tilburg University, PO Box 90153, 5000 LE Tilburg, The Netherlands

**Keywords:** Behavior change, Physical activity, Prevention, Commitment devices, Behavioral economics, Deadlines

## Abstract

To overcome self-control difficulties, people can commit to their health goals by voluntarily accepting deadlines with consequences. In a commitment lottery, the winners are drawn from all participants, but can only claim their prize if they also attained their gym-attendance goals. In a 52-week, three-arm trial across six company gyms, we tested if commitment lotteries with behavioral economic underpinnings would promote physical activity among overweight adults. In previous work, we presented an effective 26-week intervention. In the present paper we analyzed maintenance of goal attainment at 52-week follow-up and the development of weight over time. We compared weight and goal attainment (gym attendance ≥ 2 per week) between three arms that—in the intervention period- consisted of (I) weekly short-term lotteries for 13 weeks; (II) the same short-term lotteries in combination with an additional long-term lottery after 26 weeks; and (III) a control arm without lottery-deadlines. After a successful 26-week intervention, goal attainment declined between weeks 27 and 52 in the long-term lottery arm, but remained higher than in the control group. Goal attainment did not differ between the short-term lottery arm and control arm. Weight declined slightly in all arms in the first 13 weeks of the trial and remained stable from there on. Commitment lotteries can support regular gym attendance up to 52 weeks, but more research is needed to achieve higher levels of maintenance and weight loss.

## Introduction

The World Health Organization has identified physical inactivity as the fourth leading risk factor for global mortality, accounting for 3.2 million deaths annually (Forouzanfar et al., 2016). While many people know that regular physical activity (PA) is beneficial for their health and can contribute to weight management (Hildebrandt et al., 2007), most Americans (79%) and Europeans (66%) do not meet recommended levels of PA (CDC, 2014; Lee et al., 2012; Eurobarometer, 2014). Besides, overweight and obese individuals generally exercise less than normal-weight individuals (CBS & RIVM, 2014) and the prevalence of obesity has more than doubled since 1980 (WHO, 2015): approximately 70% of Americans and 62% of Europeans are currently overweight or obese. Consequently, research in the field of health promotion has yielded numerous effective ways for people to change their health behaviors (Hoeymans et al., 2014). Still, most short-term interventions only have short-term effects (Van den Berg & Schoemaker, 2010). Although many people intend to improve their health by exercising on a regular basis for longer periods, the majority fails to follow through (Kooreman & Prast, 2010).

The progressing field of behavioral economics has identified systematic and predictable decision patterns that can explain why people make decisions that deviate from their own long-term health goals. Failures of self-control have been associated with present bias: the human tendency to disproportionally overweigh costs and benefits that are immediate (e.g., exercising vs. relaxing) over those that are delayed (e.g., good health in the future) (Ainslie, 1975; Laibson, 1997). Consequently, people often intend to exercise on a regular basis, but eventually attend their gym less frequently than they had planned to (DellaVigna & Malmendier, 2006; Schumacher et al., 2017). In the present work, we aimed to test whether some of the same decision-biases that contribute to unhealthy behaviors can be used to durably assist individuals who have trouble sticking to their PA goals.

There is increasing behavioral economic evidence for the notion that people who foresee their self-control troubles can benefit from -and are willing to use- interventions that are known as commitment devices (Bryan et al., 2010). Commitment devices are defined as voluntarily imposed arrangements that restrict future behavior to avoid temptation (Rogers et al., 2014). For example, people cut up their credit cards or embrace withdrawal penalties on their savings account to avoid overspending and undersaving in the future (Beshears et al., 2015; Bryan et al., 2010) or request nearby deadlines for their work to preempt procrastination (Ariely & Wertenbroch, 2002). Likewise, Dutch employees are happy with mandatory pension savings because they fear that otherwise they would not save enough for retirement (Van Rooij et al., 2007).

Psychologically grounded commitment devices have also been tested as a tool to support health behavior change (Rogers et al., 2014). Based on the principle that humans dislike losses more than they like gains of equal size (Kahneman & Tversky, 1979), individuals have agreed to forfeit their monetary deposits at voluntarily imposed deadlines if they do not quit smoking or fail to stick to their diet (Giné et al., 2010; Halpern et al., 2012; Volpp et al., 2008). Recently, lottery-based commitment devices have also been demonstrated to support weight loss, medication adherence and walking (Kimmel et al., 2012; Patel et al., 2016a, b; Volpp et al., 2008). The lotteries aimed to tap into the human tendency to avoid regret (Christy et al., 2016; Ferrer et al., 2012; Zeelenberg & Pieters, 2007) by only awarding prizes to lottery winners who attained their health goals and informing unsuccessful lottery winners on their forgone prizes.

For the present trial, we utilized previous psychological and behavioral economic knowledge to design lottery deadlines aimed at assisting overweight adults in attaining their weekly gym attendance goals. In our *commitment lotteries* we also used the guarantee of feedback by only awarding prizes to lottery winners who attained their attendance goals and informing unsuccessful lottery winners on their forgone prizes as a way to emphasize the deadlines.

As we presented in previous work (Van der Swaluw et al., 2018), in the current sample, 13 weekly lotteries (short-term lottery arm) supported regular gym attendance. After the 13 weekly lotteries, participants in our trial attended their gym considerably less. Adding an additional long-term lottery deadline after 26 weeks (long-term lottery arm) partly averted the decline in gym attendance, indicating that a long-term lottery deadline can help sustain regular gym attendance up to 13 additional weeks. The adjusted probability of goal attainment (week-gym attendance ≥ 2) in weeks 1–13 was 57% in the short-term lottery arm, 66% in the long-term lottery arm and 25% in the control arm. In both lottery arms, 8 out of the 13 weekly winners were eligible for their prize. Between weeks 14–26, the probability of week-goal attainment was highest in the long-term lottery arm (51%) versus 23% in the other arms. The majority of participants (55%) in the long-term lottery arm was eligible to claim the prize in week 26 if they would have won it.

For the present paper, we analyzed gym attendance after all lotteries ended.

Although there is evidence that commitment devices (e.g., voluntary imposed deadlines with consequences) in the health domain are effective in the short-run, the long-term effects are often unsatisfactory (John et al., 2011; Patel et al., 2016a, b; Royer et al., 2012). Consequently, one of the key challenges in the application of commitment devices is either safeguarding maintenance of behavior change after an intervention or continuing its application (Halpern et al., 2012; Rogers et al., 2014). We focused on the former and with this purpose we examined individual goal attainment (week-gym attendance ≥ 2) after the interventions.

After all deadlines had passed, gym attendance was monitored up to week 52 in all arms to examine maintenance of behavior change. Specifically, we questioned whether goal attainment differed between the long-term lottery arm, the short-term lottery arm and the control arm between weeks 27 and 52. We hypothesized that goal attainment in the lottery arms would decline between weeks 27 and 52, but still be significantly higher than goal attainment in the control arm and would be highest in the long-term lottery arm (Van der Swaluw et al., 2016).

We additionally studied weight patterns over the course of the trial. Next to –among others– genetic, sociocultural, economic and environmental factors, modifiable behaviors (diet and PA) markedly contribute to the development of overweight and obesity (Bray et al., 2016). As such, regular PA can contribute to weight loss and weight management (Fogelholm, 2010; Fogelholm & Kukkonen-Harjula, 2000). Furthermore, overweight has been associated with self-control problems (Ikeda et al., 2010; Schlam et al., 2013), while commitment lotteries aim to combat this. Hence, we also explored the effect of the interventions on the development of weight over the 52 weeks of this trial.

## Method

### Interventions

We compared two intervention arms to a control arm. Participants in all arms set the goal to exercise twice per week. Participation was free of charge and all participants were offered monthly statistics on their progress. The control arm was also actively monitored, but was neither aware of- nor participated in the lotteries. In all arms, participants were supervised as usual by the gym staff and were free to choose their preferred mode of exercise. All gyms were equipped to facilitate endurance training, strength training and standardized group classes (e.g., circuit training). As a normal part of the gym membership, participants had access to a variety of ready-to-use training schedules that fit different exercise goals (e.g., weight loss vs. enhancing stamina).

### Short-term lottery arm

Participants in the short-term lottery arm participated in 13 weekly lotteries worth €100 each of the first 13 weeks of the trial. The weekly winners were randomly drawn out of all participants in this arm and were communicated to all by email and text message. The weekly winners were only eligible for their prize if they attended their gym at least twice that week (the week goal). Of key importance was also that lottery winners who did not attain their goal were informed about their forfeited prize. If a participant won one of the weekly lotteries, but did not attain the week goal in that week, it was communicated that “you won the lottery this week, but cannot claim your prize since you did not meet your goal of attending the gym twice”. Participants were fully informed and reminded about the possibility of this counterfactual feedback and the course of the lotteries. All other candidates in this arm were informed whether the prize was granted or forfeited. In each of the 13 weeks, the winner was drawn out of all participants regardless of prior performance. Thus, every new week meant a renewal of the commitment to exercise twice a week.

### Long-term lottery arm

The first 13 weeks in the long-term lottery arm were identical to the short-term lottery arm. Participants knew prior to the trial that weeks 14–26 would also be part of the intervention. After 26 weeks a luxury family-vacation voucher was ascribed to a randomly drawn participant in this arm. Again, the winner was communicated to all participants by email and text message. Participants knew and were reminded that they would always learn the outcome of the lottery, but that the prize could only be claimed if the winner had attained his or her goal in at least 9 of the second 13 weeks (70% between weeks 14 and 26). We guaranteed that the prize would eventually be awarded: if the winner was not eligible for the prize, he or she would be informed of the forgone prize and another winner would be drawn until the prize could be awarded according to the rules mentioned above.

### Design and setting

**Fig. 1:**
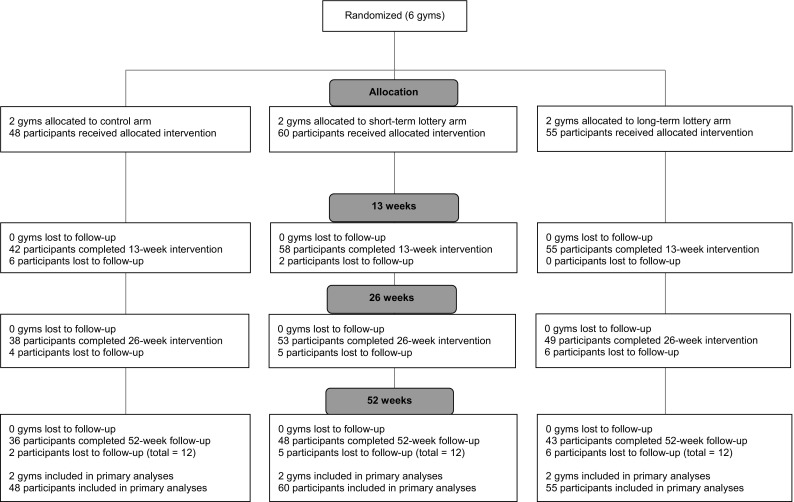
Study design and flow of gyms and participants

The study design and details on randomization, blinding, eligibility, recruitment and measurement protocols have been published before (Van der Swaluw et al., 2016). In brief, we set up a three-arm, parallel group, cluster randomized trial running for 52 weeks with 163 participants in six company gyms (clusters) across the Netherlands. Figure 1 displays the trial flow over 52 weeks. The gyms were branches of international fitness agency High Five, which provides corporate fitness training in 36 organizations across the Netherlands. The trial was reviewed and approved by the Tilburg University Ethical Review Board (EC-2014.42a) and is registered in the Dutch Trial Register (NTR5559). The lottery drawings were performed by the independent Game Management Department of the Dutch State Lottery under supervision of a notary.

The six gyms were randomized to one of three arms. As such, every arm contained two gyms. Participants were eligible if they expressed in a survey their goal to exercise twice or more per week, were between the ages of 18–65, were overweight (25 ≤ BMI < 40) and had not planned a leave of absence of more than 4 weeks in the first 26 weeks of the trial. Participants were blinded from the other trial arms. Informed consent was obtained from all individual participants included in the study. Table 1 displays the baseline data of participants in all three arms.

**Table 1:** Participant demographics per study arm

Characteristic	Control (*n* = 48)	Short-term lotteries (*n* = 60)	Long-term lottery (*n* = 55)
Age, mean (SD)	50 (9.84)	49.3 (9.33)	45 (9.58)
Gender, no. (%)			
Female	16 (33.3)	21 (35)	13 (23.6)
Male	32 (66.7)	39 (65)	42 (76.4)
No survey response, no. (%)	3 (6.25)	0 (0)	1 (1.67)
Nationality, no. (%)			
Dutch	36 (80)	52 (86.7)	52 (96.3)
Other	12 (20)	8 (13.3)	3 (3.7)
Education, no. (%)			
Pre-vocational education	3 (7.9)	7 (11.5)	4 (7.4)
Pre-university education	3 (6.7)	2 (3.3)	10 (18.5)
Senior vocational training	11 (24.4)	20 (33.3)	5 (9.3)
Vocational colleges	19 (42.2)	15 (25)	23 (42.6)
University education	9 (20)	15 (25)	10 (18.5)
Other	0 (0)	1 (1.7)	2 (3.7)
Monthly net income, no. (%)			
< €1000	0 (0)	0 (0)	1 (1.8)
€1000 to €2000	10 (20.8)	6 (10)	3 (5.5)
€2000 to €3000	19 (39.6)	32 (53.3)	24 (43.6)
€3000 to €4000	8 (16.7)	15 (25)	19 (34.5)
€4000 to €5000	2 (4.2)	1 (1.7)	2 (3.6)
€5000 tot €6000	0 (0)	2 (3.3)	1 (1.8)
> €6000	1 (2.1)	0 (0)	0 (0)
Did not wish to answer	5 (10.4)	4 (6.7)	4 (7.3)
Baseline gym attendance^a^, mean (SD)	1.82 (0.88)	1.46 (1.17)	1.55 (1.04)
Weight, mean (SD)	90.14 (14.38)	96.12 (14.12)	96.6 (13.94)
BMI, mean (SD)	28.9 (3.20)	30.4 (3.73)	30.19 (3.47)
Obese, no. (%)	13 (27.1)	23 (38.3)	26 (47.3)

^a^Participants answered the question; “On average, how often per week did you attend the gym in the last 2 months?”

### Outcomes and measures

Of primary interest in this trial was goal-attainment (week-gym attendance ≥ 2) measured at the participant level. We provided all gyms with an iPad connected via Wi-Fi, which allowed us to monitor attendance in real time. Throughout the 52 weeks of the trial, participants were required to check in at the iPad with their name or three digit study-ID when entering their gym. All gyms were provided with identical scales to measure weight (KERN™; 0.1% precision). Upon registering their attendance, participants were asked to weigh (without shoes) and to enter their weight (kilograms, 1 decimal) into the iPad. Participants could also select the option; “I already entered my weight this week”, or “I will enter my weight later this week”. Hence, weight was assessed on a weekly basis. At baseline, 13-, 26- and 52 weeks, participants were supervised by the gym personnel in entering their weight. Baseline attendance levels and demographics were assessed via an additional online questionnaire.

### Analyses

Participants were the primary unit of inference in all analyses. Analyses followed the intention-to-treat principle and were conducted in R version 3.4.0 with statistical significance set at *p*  <  0.05. Planned analyses can also be found in the trial protocol (Van der Swaluw et al., 2016).

To evaluate the effect of the interventions on goal attainment per week, we performed three multi-level logistic regression analyses with goal attainment from weeks 1–13, 14–26, and 27–52 as the dependent variable respectively. These measurements are nested within participants who are clustered within gyms. The three trial-arms, time, self-reported baseline attendance, age and sex were entered as fixed effects. Random intercepts were added for both the participants and the gyms. A random slope for time on the participant level was also included in the model and hereby allowed for different time patterns among individuals. Week 42 of the trial was excluded from the analyses, because gyms were closed in that week as a result of the new-year holiday season.

When there are few gyms per arm, treatment effects may correlate with intra cluster (gym) effects. By adding the random intercept for gyms, the model estimated the treatment effects, while accounting for the clustered data pattern in gyms. Additionally, sensitivity analyses were performed by excluding each gym from the models once and comparing effects of the reduced data models to the full model.

To assess the effect of the interventions on weight in each trial-period, multi-level linear regression analyses were performed with weight (kilograms) as the dependent variable. Linear multi-level modelling has shown to be a reliable technique to handle missing longitudinal outcome data (Peters et al., 2012) and was used to fit weight patterns over time, despite missing outcome measurements of participants who did not enter their weight that week. As before, these measurements are nested within participants who are clustered within gyms. Again, random intercepts were added for both the participants and the gyms. A random slope on the participant level was also added. The three trial-arms, time, baseline BMI, age and sex were included in all models as fixed effects.

## Results

### Goal attainment

**Fig. 2:**
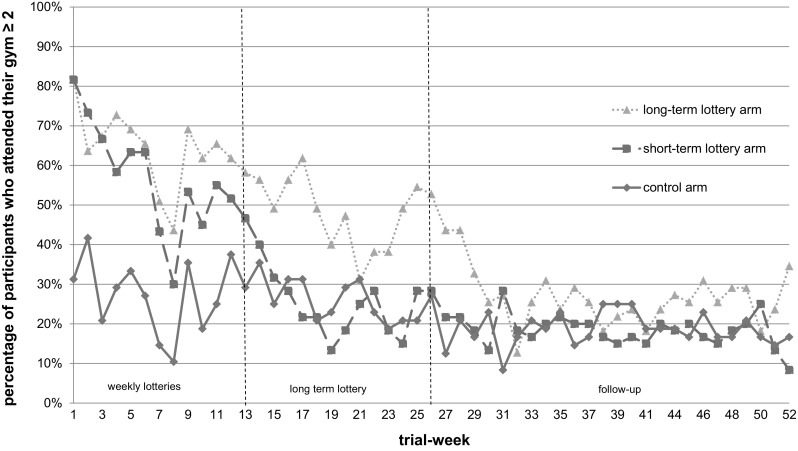
Goal attainment (week-gym attendance ≥ 2) over time per arm

Figure 2 displays percentages of goal attainment per arm per week. Results on goal-attainment in the first 26 weeks of the trial have been presented before (Van der Swaluw et al., 2018). Between weeks 27- 52, the aggregated percentage of goal attainment was 24% in the long-term lottery arm and 16 and 15% in the short-term arm and control arm respectively. Table 2 displays the time-adjusted odds ratios of goal attainment in each trial period.

**Table 2:** Logistic mixed models describing goal attainment (week-attendance ≥ 2)

	Weeks 1–13	Weeks 14–26	Weeks 27–52
	Odds ratio (95% CI)	Odds ratio (95% CI)	Odds ratio (95% CI)
Trial characteristics			
Control arm	(ref.)		
Short-term lotteries	12.10** (2.54–57.53)	1.15 (0.23–5.73)	1.84 (0.30–11.54)
Long-term lottery	13.47** (2.76–65.74)	6.13* (1.18–31.92)	7.88* (1.18–52.51)
Time (week)	0.93 (0.86–1.00)	0.92 (0.84–1.02)	0.96 (0.91–1.01)
Short-term × time	0.92 (0.84–1.01)	1.01 (0.90–1.13)	0.99 (0.94–1.04)
Long-term × time	1.01 (0.91–1.11)	1.01 (0.91–1.11)	0.97 (0.92–1.03)
Participant characteristics			
Baseline attendance	1.39* (1.05–1.85)	1.92*** (1.32–2.80)	2.35* (1.40–3.93)
Age	1.00 (0.97–1.03)	1.04 (1.00–1.09)	1.05 (0.99–1.11)
Male (ref.) vs. Female	0.39** (0.21–0.75)	0.61 (0.26–1.43)	0.76 (0.22–2.58)

Model accounts for clustered measures within gyms, participants and for temporal trends by week. Outcome is a binary term (0 or 1)

*CI* confidence interval, *Ref* reference category

*Significant at *p* < 0.05

**Significant at *p* < 0.01

***Significant at *p* < 0.001

In the logistic mixed model fitting weekly goal attainment between weeks 27–52, the long-term lottery arm had significantly higher odds of goal attainment than the control arm (OR 7.88, 95% CI 1.18–52.51, *p* = 0.03) and non-significant higher odds than the short-term lottery arm (OR 4.31, 95% CI 0.83–22.36, *p* = 0.08). The difference in goal attainment between the short-term lottery arm and the control arm was not statistically significant (OR 1.84, 95% CI 0.30–11.54, *p* = 0.15).

### Sensitivity analyses

The intervention effect between weeks 27 and 52 was only sensitive to the sequential exclusion of control gyms from the models. If we excluded the best performing gym from the control arm, the intervention effect of the short-term lottery arm became statistically significant. If we excluded the least performing gym from the control arm, the effect of the long-term lottery arm remained qualitatively similar, but was no longer statistically significant.

### Weight

Table 3 displays the output of the linear mixed models describing weight in each trial period. In the first 13 weeks of the trial, weight declined slightly over time in the control arm (*B*: − 0.09, SE: 0.03, *p* = 0.002), the short-term lottery arm (*B*: − 0.08, SE: 0.02, *p* = 0.001) and the long-term lottery arm (*B*: − 0.06, SE: 0.03, *p* = 0.02). The decline in weight did not differ between arms. Between weeks 14–26 and weeks 27–52, the models display neither a significant decline of weight over time, nor significant differences between arms.

**Table 3:** Linear mixed models describing the weight (kilograms)

	Weeks 1–13		Weeks 14–26		Weeks 27–52	
	Unstandardized Beta (SE)	*t* value	Unstandardized Beta (SE)	*t* value	Unstandardized Beta (SE)	*t* value
Trial characteristics						
Control arm	− 1.02 (5.47)	− 0.19	− 0.19 (6.26)	− 0.03	− 1.33 (6.79)	− 0.20
Short-term lotteries	0.90 (1.85)	0.49	− 0.05 (1.86)	− 0.03	− 0.63 (2.08)	− 0.30
Long-term lottery	1.40 (1.92)	0.73	1.11 (1.94)	0.57	0.44 (2.13)	0.21
Time (week)	− 0.09 (0.03)**	− 3.03	− 0.05 (0.03)	− 1.41	− 0.01 (0.04)	− 0.28
Short-term × time	0.01 (0.04)	0.31	0.04 (0.05)	0.93	0.01 (0.05)	0.20
Long-term × time	0.03 (0.04)	0.80	0.05 (0.04)	1.14	− 0.01 (0.05)	− 0.14
Participant characteristics						
Baseline BMI	2.84 (0.18)***	15.87	2.79 (0.21)***	13.60	2.89 (0.22)***	12.94
Age	0.07 (0.07)	1.05	0.09 (0.08)	1.22	0.11 (0.09)	1.21
Male (ref.) vs. Female	13.73 (1.39)***	9.89	14.06 (1.54)***	9.16	13.13 (1.74)***	7.57

Model accounts for clustered measures within gyms, participants and for temporal trends by week

*SE* Standard Error, *Ref* reference category

*Significant at *p* < 0.05

**Significant at *p* < 0.01

***Significant at *p* < 0.001

## Discussion

The 52-week follow-up analyses of our cluster randomized trial show moderately sustained levels of goal attainment (gym week-attendance ≥ 2) six months after completing a 26-week lottery intervention. Up to one year after the start of the intervention, participants who entered 13 weekly commitment lotteries (weeks 1–13), followed by an additional lottery 13 weeks later (weeks 14–26) were more likely to attend their gym twice per week than participants in the control arm. In the same follow-up period, goal attainment in the short-term lottery arm (13 weekly commitment lotteries) did not differ from the control arm or long-term lottery arm. Weight declined slightly in the first 13 weeks of the trial in all arms and remained stable from there on.

The present study may contribute to the pursuit of methods for sustainable behavior change. Commitment lotteries have been used relatively sporadically in the field of health promotion. Starting a decade ago, Volpp et al. (2008) have offered lotteries to overweight participants to support their weight loss attempts. While their lotteries were effective for 16 weeks, participants regained weight after the intervention. Likewise, Patel et al. (2016a, b) have effectively used team-based lotteries to stimulate walking for 13 weeks, but differences between intervention and control deteriorated during the 26-week follow-up period. Although there is evidence for their short-term effectiveness, the longevity of commitment lotteries is unsatisfactory. In the present trial, we observe a significantly higher likelihood of goal attainment in the long-term lottery arm than control in the six months after the intervention. The observation that the long-term lottery-arm does not underperform the control arm during follow-up implies that the 26-week intervention has had a net effect on gym attendance over a 1-year period.

Still, in the interpretation of our results, some nuance is warranted. In Fig. 2 it can be observed that levels of goal attainment in the long-term lottery arm dropped after the 26-week lottery deadline. Accordingly, average week-goal attainment in the long-term lottery arm halved during follow-up, relative to weeks 14–26. Therefore, it cannot be concluded that participants in the long-term lottery arm remained equivalently committed to their gym attendance goal as before. Similar to previous studies, behavior change is shown to be challenging to maintain after commitment lotteries end.

Nonetheless, the tested combination of short-term lotteries and a long-term lottery in this trial provides novel and useful insights that can be built upon in future trials to help optimize the long-term effectiveness of commitment lotteries. For example, for the winner to be eligible for the long-term lottery prize, he or she had to attain at least 9 week-goals between weeks 14–26. The majority of participants in the long-term lottery arm did so. Therefore, future studies can experiment by lengthening the long-term lottery deadline (e.g., to 36 weeks (John et al., (2011) to test if this will further stimulate sustained goal attainment. This exploration may result in a deadline that is nearby enough to be salient in the present, while being lengthy enough to promote sustained behavior change after the deadline.

Similar to previous research, behavioral economic and psychological insights on decision-making could be well incorporated in health promotion (Loewenstein et al., 2012). First, to leverage present bias (the overweighing of the present), we imposed nearby deadlines to draw the consequences of procrastination nearer (Ariely & Wertenbroch, 2002). Second, because people tend to overestimate small probabilities, a lottery is an effective and scalable tool to make missing the deadlines potentially costly for participants (Kahneman & Tversky, 1979). Third, we leveraged the human tendency to avoid regret by drawing the lottery prize from all participants and informing non-eligible winners on their forgone prize (Zeelenberg & Pieters, 2004, 2007). Fourth, while we aimed to stimulate long-term behavior change, we used week-goals to fit individuals’ impatience and to facilitate the human tendency of using of temporal landmarks (e.g., Mondays) to relegate misfortune to the past and have a fresh start (Dai et al., 2014; O’Donoghue & Rabin, 1999).

The results of this trial also offer several practical insights for health professionals, policy makers, insurers or employers who aim to support people in achieving their health goals. Next to its effectiveness, the costs of prevention are considered a key aspect in the determination of its value to businesses and society (Van den Berg & Schoemaker, 2010). The use of psychological and behavioral economic insights in the design of the lotteries can enhance the psychological impact of money that is spent and could offer the opportunity to implement commitment lotteries at low costs. To stimulate regular gym attendance up to 52 weeks in the long-term lottery arm, we spent only €2.21 per participant per week (awarded prizes ÷ participants ÷ weeks). In perspective; this is 0.6% of the Dutch minimum week-wage (€361.25). The costs in future applications may be further reduced if participants are also willing to pay for their wish to commit, similar to self-imposed withdrawal penalties on savings accounts (Beshears et al., 2015) or betting one’s money on personal health goals with a deposit contract (Halpern et al., 2012). It would be valuable to explore the characteristics of participants and potential organizers of commitment lotteries that contribute to the optimal balance between attractiveness and effectiveness.

Despite their potential, commitment devices remain underused (Halpern et al., 2012; Rogers et al., 2014)- a fact that might be explained by the range of open issues on their implementation. For example, employees or patients outside the interventions may resent others receiving lottery prizes (Loewenstein et al., 2012) (which may also be circumvented by asking participants to pay for lottery tickets). Besides, commitment lotteries were especially effective while they were active and -analogous to drugs- lost most of their effectiveness once people stopped ‘taking them’. This finding stimulates thinking about the type of commitment device that maintains its impact over repeated (and possibly infinite) application. While goal attainment declined over time in our trial (see Fig. 2), the decline of goal attainment over time did not differ significantly between the lottery arms and the control arm. Therefore, it would be interesting to design and test similar commitment lotteries that, like many commercial lotteries, are endlessly repeated and accessible.

Another issue surrounding the applicability of commitment lotteries is their target population. The majority of our sample was male (69.3%), which was similar to the general population of the six gyms (69.8% male). Nonetheless, future studies would benefit from enrolling an even proportion of females and males. A meta-analysis by Haff et al. (2015) that compared the effectiveness of several commitment lotteries between demographic groups found no gender differences, nor differences in education or broad ranges of income (except a slight reduced effect with incomes > $87.500). In previous analyses, we also observed no differences in intervention-effectiveness between income- or education categories (Van der Swaluw et al., 2018). This may be an indication of commitment lotteries being broadly applicable. A next step in the development of commitment devices could be the assessment of design features that contribute to their efficacy and feasibility across different populations. Hence, although there is increasing evidence for the effectiveness of commitment lotteries, more applied research could enhance their wide-spread practicability.

Commitment lotteries aimed at promoting regular PA did not result in substantial weight loss. After a moderate decline in weeks 1–13, weight remained stable over time in all arms, which is contrary to projections of overweight and obesity progressing over time (Wang et al., 2008). Considering that medical complications and associated health care costs rise progressively as BMI increases (Cawley & Meyerhoefer, 2012), stable weight is not all bad. Nonetheless, no meaningful weight loss was achieved as a result of increased PA, meaning that most participants remained exposed to the increased risks of cardiovascular diseases, diabetes type 2 and cancers that are associated with overweight and obesity (Forouzanfar et al., 2016; WHO, 2013). Our findings are in line with the general conception that regular exercise can contribute to weight loss but often not solely (Fogelholm, 2010; Fogelholm & Kukkonen-Harjula, 2000). In some occasions, increases in PA have also been found to result in weight gain by calorie compensation (McCaig et al., 2016).

To achieve significant and sustained weight loss, regular PA is only one part of the multicomponent (lifestyle training, nutrition and PA) interventions that are acknowledged to be effective (Bray et al., 2016). Still, weight loss interventions that include an exercise component are often more effective than interventions that do not (de Roon et al., 2017; Jeffery et al., 2003). Given that overweight individuals generally exercise less than normal weight individuals (CBS & RIVM, 2014), any multicomponent weight loss intervention that also encompasses PA might still benefit from including commitment lotteries for PA.

This trial is subject to several limitations. First, while it is clear that commitment lotteries did not promote meaningful weight loss, it remains unclear why this was the case in this trial (e.g., due to the type of exercise, calorie compensation, loss of fat that may have been compensated by increased muscle mass). We chose to focus on gym attendance as our main dependent variable, rather than weight loss, because this is under more direct volitional control by the participants. In future instances, it may be valuable to explore the need for additional weight loss guidance when participants enter commitment lotteries for PA. A second limitation is that our trial limited the promotion of regular PA to gym attendance, while activities with lower intensity (e.g., walking and recreational cycling) are also known to promote health outcomes (Fogelholm, 2010). The benefit of our approach is the novel and scalable context in which commitment lotteries were shown to be effective (company gyms), while exercise was supervised by the gym personnel. Third, our trial included no more than six gyms and randomization at the gym level increased the potential influence of intra gym effects in the effectiveness of the interventions. The sensitivity analysis (where we excluded the best performing control-gym from the model) reduced the sustained effect of the long-term lottery to non-significance. We accounted for intra-gym influences in our multi-level analyses and randomization at the gym level had the benefit that treatment contamination was minimized. Still, future studies can potentially avoid these issues by enrolling more gyms.

## Conclusion

Regular PA has numerous health benefits. While many people aim to exercise on a regular basis, there are multiple predictable behavioral patterns that hamper the progression of a health-intention to sustained behavior. Effective and scalable support of long-term regular exercise is needed. Commitment lotteries that were designed to leverage psychological knowledge on decision-making can help cope with the challenges of health behavior change at low costs. While the 26-week intervention supported regular PA up to 52 weeks, levels of gym attendance declined after all deadlines had passed. Participants did not remain equivalently committed as before the long-term lottery deadline. Future research in broader populations could reveal to what degree commitment lotteries remain effective over longer time periods or if they are endlessly accessible.
